# Single-cell transcriptomics reveals cellular heterogeneity and molecular stratification of cervical cancer

**DOI:** 10.1038/s42003-022-04142-w

**Published:** 2022-11-10

**Authors:** Chunbo Li, Hao Wu, Luopei Guo, Danyang Liu, Shimin Yang, Shengli Li, Keqin Hua

**Affiliations:** 1 Department of Obstetrics and Gynecology, Obstetrics and Gynecology Hospital of Fudan University, Shanghai, China; 2 Precision Research Center for Refractory Diseases, Institute for Clinical Research, Shanghai General Hospital, Shanghai Jiao Tong University School of Medicine, Shanghai, China; 3 Laboratory of Molecular Neural Biology, School of Life Sciences, Shanghai University, Shanghai, 200444 China; 4 Department of Pathology, Obstetrics and Gynecology Hospital of Fudan University, Shanghai, China

**Keywords:** Cervical cancer, Data mining, Cancer microenvironment

## Abstract

Cervical cancer (CC) is the most common gynecological malignancy, whose cellular heterogeneity has not been fully understood. Here, we performed single-cell RNA sequencing (scRNA-seq) to survey the transcriptomes of 57,669 cells derived from three CC tumors with paired normal adjacent non-tumor (NAT) samples. Single-cell transcriptomics analysis revealed extensive heterogeneity in malignant cells of human CCs, wherein epithelial subpopulation exhibited different genomic and transcriptomic signatures. We also identified cancer-associated fibroblasts (CAFs) that may promote tumor progression of CC, and further distinguished inflammatory CAF (iCAF) and myofibroblastic CAF (myCAF). CD8^+^ T cell diversity revealed both proliferative (*MKI67*^+^) and non-cycling exhausted (*PDCD1*^+^) subpopulations at the end of the trajectory path. We used the epithelial signature genes derived from scRNA-seq to deconvolute bulk RNA-seq data of CC, identifying four different CC subtypes, namely hypoxia (S-H subtype), proliferation (S-P subtype), differentiation (S-D subtype), and immunoactive (S-I subtype) subtype. The S-H subtype showed the worst prognosis, while CC patients of the S-I subtype had the longest overall survival time. Our results lay the foundation for precision prognostic and therapeutic stratification of CC.

## Abstract (web summary)

Single-cell RNA sequencing of cervical cancer reveals four different cancer subtypes and provides information on populations of cancer-associated fibroblasts as well as CD8+ T cell diversity.

## Introduction

Cervical cancer (CC) is one of the most frequent female malignancies around the world^1^. CC ranked the fourth of incidence and the fourth of mortality across all cancer types in women^2^. According to the World Health Organization (WHO), an estimated 604,000 new cases and 342,000 deaths of CC were reported around the world in 2020. A large proportion of CC cases are reported to be related with the human papillomavirus (HPV)^3^. Accompanying the HPV infections, some genetic factors contribute a lot to the development of CC^4^. Although patients with early CC can survive for years after surgery or radiation therapy, those diagnosed with advanced or metastasized CC is incurable. Therapeutics against advanced or recurrent CC are available, such as anti-angiogenesis and immunotherapy, but the response rate is still low^5,6^, which is mainly due to the inter-tumor and intra-tumor heterogeneity of CC. Therefore, understanding the heterogeneity of CC in high resolution is crucial for the development of personalized therapeutic strategies.

The Cancer Genome Atlas (TCGA) group reported a comprehensive molecular characterization of CC by profiling genomics, transcriptomics and proteomics in 288 CC samples^7^. They revealed highly heterogenous molecular profiles across samples and identified three subtypes, i.e., the keratin-low squamous, keratin-high squamous and adenocarcinoma-rich subtype. Zhu et al. identified two subtypes of HPV^+^ CC based on the most varied 50 genes across CC samples^8^. Furthermore, an increasing number of studies have identified molecular units (including DNA, RNA, and proteins) as biomarkers for the diagnosis and treatment of CC^9–11^. But these studies were based on bulk sequencing data, thus overlooking the extensive cellular heterogeneity of CC. Recently, our study based on single-cell RNA sequencing (scRNA-seq) provided a glimpse into the phenotypic diversity and ecosystems of CC microenvironment^12^. In this study, we presented a comprehensive characterization of CC cellular heterogeneity by utilizing scRNA-seq. We identified subpopulations of epithelial cells, fibroblasts, and CD8^+^ T cells, illustrating the cellular heterogeneity of CC. Based on the signature genes derived from scRNA-seq analysis, we identified four different CC subtypes that exhibited clinical significance. Our study shed light on the cellular heterogeneity and promoted the personalized treatment of CC.

## Results

### Single-cell transcriptomics analysis reveals extensive heterogeneity of malignant cells in human CC

**Fig. 1:**
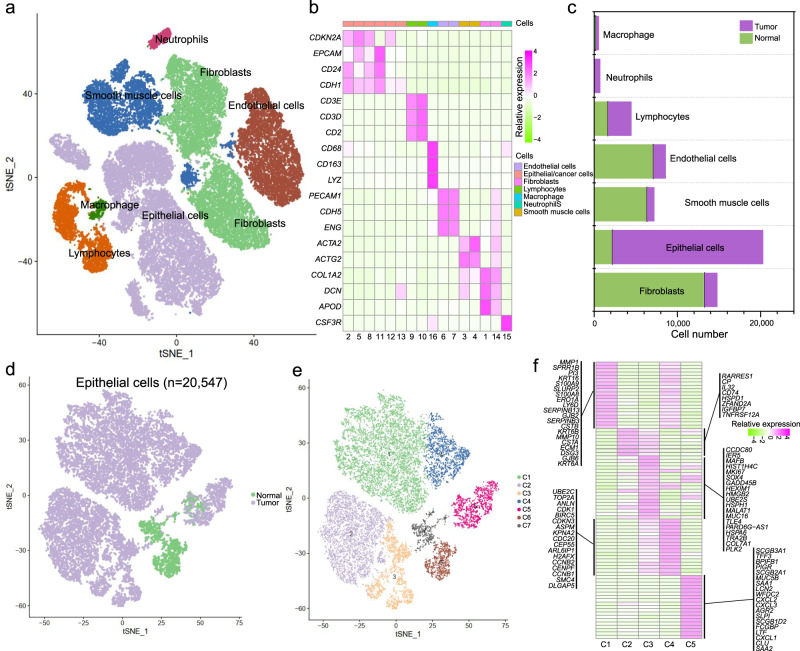
Tumor heterogeneity of CC at single-cell resolution. **a** UMAP dimensionality reduction of all cells. **b** Heatmap shows the relative expression of top marker genes in each cell type. **c** The cell numbers in CC tumor and NAT samples for each cell type. **d** UMAP shows epithelial cells in CC tumor and NAT samples. **e** UMAP shows seven different clusters of epithelial cells. **f** Heatmap shows the top marker genes in each epithelial subcluster.

To investigate the cellular diversity and distinct molecular signatures in CC, scRNA-seq was performed in three CC cancer and paired NAT samples (Supplementary Fig. 1a). A total of 57,669 cells were obtained after stringent filtering, with specific cell groups of tumor or NAT samples (Supplementary Fig. 1b). These cells were further classified into 16 different clusters (Supplementary Fig. 1c). Marker genes in each cluster were then compared to known markers of cervical cells to determine known cell types (see “Methods”). These 16 cell clusters were assigned to seven different cell types (Fig. 1a), including epithelial cells (20,547 cells, 35.6%, marked with *CDKN2A*, *EPCAM*, *CD24*, and *CDH1*), endothelial cells (8617 cells, 14.9%, marked with *PECAM1*, *CDH5*, and *ENG*), fibroblasts (15,304 cells, 26.5%, marked with *COL1A2*, *DCN*, and *APOD*), smooth muscle cells (7429 cells, 12.9%, marked with *ACTA2* and *ACTG2*), lymphocytes (4490 cells, 7.8%, marked with *CD3E*, *CD3D*, and *CD2*), macrophage (571 cells, 1.0%, marked with *CD68*, *CD163*, and *LYZ*), and neutrophils (741 cells, 1.3%, marked with *CSF3R*) (Fig. 1b). In our scRNA-seq data, the majority of smooth muscle cells (6463 cells, 87.0%) and endothelial cells (7118 cells, 82.6%) were derived from normal samples, while most epithelial cells (18,362 cells, 89.4%) were from tumor samples (Fig. 1c). Epithelial cells from tumor samples showed distinct transcriptional features with those from normal samples (Fig. 1d). Compared to those in NAT samples, both non-immune (Supplementary Fig. 2) and immune cell types (Supplementary Fig. 3) showed hundreds of differentially expressed genes that were enriched in specific pathways. All epithelial cells were further classified into seven different subclusters (Fig. 1e). Cells in subcluster C1, C2, C3, C4, and C5 were mainly from tumor samples, whereas those in the C6 and C7 subclusters were from normal samples. Cells in subcluster C1 were characterized by high expression levels of *MMP1*, *SPRR1B*, *KRT16*, *CSTA*, and *S100A9* (Fig. 1f, Supplementary Table 2). Cells in subcluster C2 exhibited high expression levels of immune-associated genes, such as *CD74*, and *IL32*. The C3 subcluster showed high expression of *CCDC80*, *IER5*, and *MAFB*. The C4 subcluster showed high expression of *UBE2C*, *TOP2A*, and *ANLN*. Subcluster C5 exhibited high expression levels of normal epithelial markers, such as *CLU*, *SCGB3A1*, and *MUC5B*. In summary, our single-cell transcriptomics analysis revealed cellular heterogeneity of cervical epithelial cells.

### CC epithelial subpopulations exhibited genomic and transcriptomic differences

**Fig. 2:**
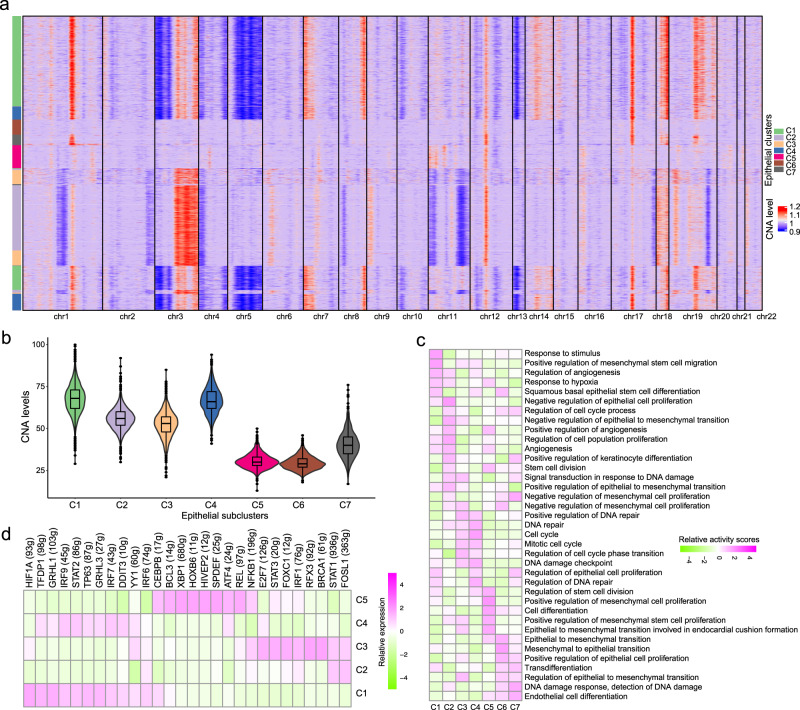
Molecular differences among epithelial cell subclusters. **a** Inferred CNA levels in each epithelial cell cluster across 22 chromosomes. **b** Comparisons of overall CNA levels among different epithelial subclusters. **c** Heatmap shows the relative activity scores of biological processes in each epithelial subcluster. **d** Heatmap shows the relative expression of differential transcription factors in each cancer epithelial cell subcluster.

To further investigate the distinctions between the identified epithelial subpopulations, we inferred the copy number aberration (CNA) of each cell based its gene expression profile (see Methods). To evaluate the malignancy of identified epithelial subclusters, analysis of CNA levels in each cell population were performed according to average expression patterns across intervals of the genome. Remarkably, subcluster C1, C2, C3, and C4 exhibited copy number gains in chromosome 3q and 18, whereas they showed copy number loss in chromosome 3p, 5, and 13 (Fig. 2a). Overall, cells from subcluster C5, C6, and C7 showed low CNA levels, while those from cluster C1, C2, C3, and C4 showed relatively high CNA levels (Fig. 2b, Supplementary Data 1). The low CNA level in subcluster C5, which is mainly from tumor samples, might indicate a well differentiated state (Fig. 1d–f). Our enrichment analysis of high expression genes revealed the enrichment of response to stimulus, response to hypoxia, regulation of angiogenesis and positive regulation of mesenchymal stem cell migration, suggesting that cells in cluster C1 acquired a malignancy behavior (Fig. 2c and Supplementary Fig. 4a). Highly expressed genes in subcluster C2 were enriched in negative regulation of epithelial stem cell proliferation, regulation of cell cycle process and regulation of cell population proliferation (Fig. 2c and Supplementary Fig. 4b). The C3 and C4 subclusters shared similar enrichment of DNA repair, cell cycle, regulation of cell cycle phase transition and DNA damage checkpoint (Fig. 2c, Supplementary Fig. 4c, d). Highly expressed genes in subcluster C5 were enriched in the regulation of stem cell division and cell differentiation (Fig. 2c and Supplementary Fig. 4e). The C6 and C7 subclusters shared the same enriched biological process of normal epithelial biology, such as epithelial to mesenchymal transition (EMT) and positive regulation of epithelial cell proliferation (Fig. 2c, Supplementary Fig. 4f, g). Next, we employed the single-cell regulatory network inference and clustering (SCENIC) method to identify transcription factors that play important regulatory roles in malignant epithelial subclusters. Our analysis revealed many transcription factors in epithelial subclusters, such as *HIF1A*, *TFDP1*, and *GRHL1* in subcluster C1, *STAT1* and *FOSL1* in subcluster C2, and *XBP1* and *NFKB1* in subcluster C5 (Fig. 2d, Supplementary Table 3). Hypoxia-induced factor-1 (HIF-1) is the most critical gene in hypoxic response and is responsible for the upregulation of many downstream effector genes that were collectively known as hypoxia-responsive genes (such as *VEGFA*, *EGF*, p53, *GLUT1*, and *GLUT3*). These genes govern multiple key biological pathways such as proliferation, energy metabolism, invasion, and metastasis. For example, as the key member of interferon signaling, *STAT1* modulates the response to intracellular and extracellular stimulation^13^. *STAT1* has been demonstrated to act as a tumor suppressor in many cancer types^14,15^. Collectively, our analysis revealed genomic and transcriptomic distinctions among epithelial subpopulations.

### Tumor-derived fibroblasts exhibited transcriptional alterations in CC

**Fig. 3:**
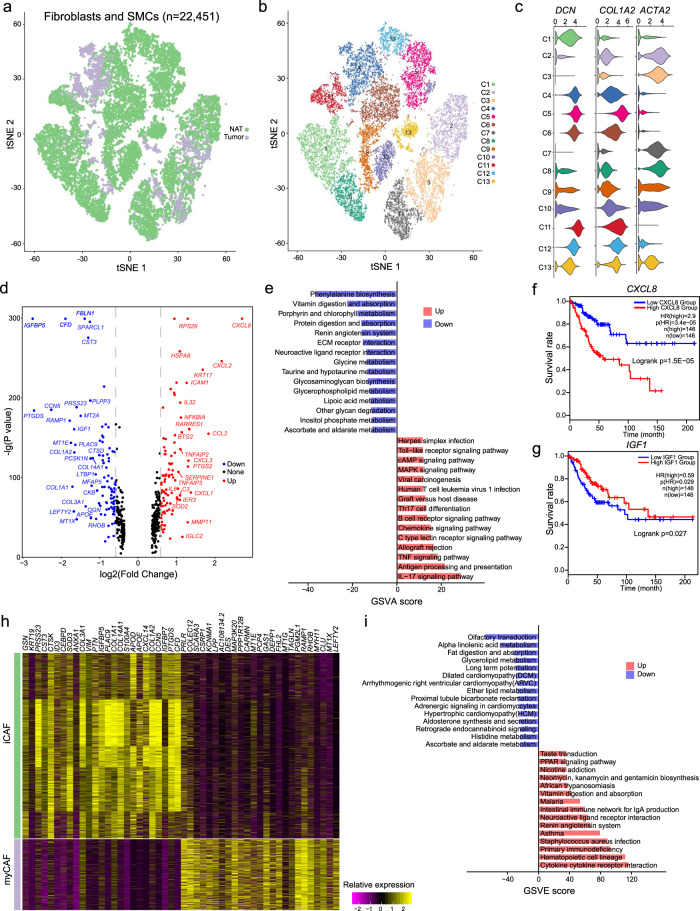
Cellular and molecular heterogeneity of fibroblasts in CC. **a** tSNE plot showing fibroblasts and SMCs in tumor and NAT cervical samples. **b** tSNE plot showing fibroblast subclusters. **c** Relative expression levels of the *DCN*, *COL1A2*, and *ACTA2* gene across 13 fibroblast/SMC subclusters. **d** Volcano plot showing gene expression differences between tumor and NAT fibroblasts. **e** Bar plots showing the relative activities of upregulated and downregulated biological processes in tumor fibroblasts. **f** The Kaplan–Meier survival curve of *CXCL8* in the TCGA cervical cancer cohort. **g** The Kaplan–Meier survival curve of *IGF1* in the TCGA cervical cancer cohort. **h** Heatmap shows differential genes between iCAF and myCAF cells. **i** Bar plots showing the relative activities of upregulated and downregulated biological processes.

We next investigated the non-immune cells within the tumor microenvironment (TME), including fibroblasts and smooth muscle cells (SMCs). We identified a total of 22,451 fibroblasts and SMCs. Most of these cells were from the cervical NAT samples (Fig. 3a). These cells were then re-clustered based on gene expression profiles, which generated 13 different clusters (Fig. 3b). These cell clusters showed different expression of marker genes *DCN*, *COL1A2*, and *ACTA2* (Fig. 3c). According to the specific cell markers, we assigned cluster C1, C4, C5, C6, C11, and C12 as fibroblasts and cluster C2, C3, C7, C8, C9, C10, and C13 as SMCs. To further explore how fibroblasts impact CC tumor progression, we examined the transcriptional alterations of tumor-derived fibroblasts. Compared to fibroblasts from normal tissues, the top upregulated genes in tumor-derived fibroblasts were *CXCL8*, *CXCL2*, *CCL2*, *CXCL3* and *CXCL1*, and the top downregulated genes were *IGFBP5*, *PTGDS*, *CCN5*, *CFD*, and *RAMP1* (Fig. 3d, Supplementary Data 2). Functional enrichment analysis revealed that fibroblasts in tumor tissue were associated with IL-17 signaling pathway, antigen process and presentation and INF-signaling pathway, indicating a potential role in immune regulation (Fig. 3e). More importantly, we identified several DEGs in fibroblasts that were significantly associated with patient prognosis, such as *CXCL8* and *IGF1*. The *CXCL8* gene was upregulated in tumor fibroblasts and the high expression was associated with poor prognosis (Fig. 3f). The *IGF1* gene was downregulated in tumor fibroblasts and the low expression was associated with poor prognosis (Fig. 3g). The *CXCL8* gene showed oncogenic, while *IGF1* exhibited tumor suppressor features in tumor fibroblasts.

In our scRNA-seq dataset, cluster C4, C5, C6, C11, and C12 fibroblasts had high expression of gene *IL6*, *IL8*, *CXCL1*, *CXCL2*, *CCL2*, and *CXCL12*, and was identified as inflammatory cancer-associated fibroblasts (iCAFs). Cluster C1 fibroblasts was identified as myofibroblastic CAFs (myCAFs) with the high levels of SMA (encoded by gene *ACTA2*). To explore the functional differences between these two fibroblast types, we identified a set of significant DEGs between iCAFs and myCAFs, including the top upregulated genes, such as *CXCL14*, *IFGBP7*, *PTGDS*, *CFD* and *CCN5*, and top downregulated genes, such as *LEFTY2*, *MT1X*, *CLU*, *MYH11*, and *RHOB* (Fig. 3h, Supplementary Data 3). Functional enrichment analysis revealed upregulated activities of immune-related biological processes, including cytokine-cytokine receptor interaction, primary immunodeficiency, and intestinal immune network for IgA production (Fig. 3i). We further compared the gene expression between cancer cells and tumor-derived fibroblasts. Differential gene analysis revealed 319 genes upregulated in tumor-derived fibroblasts, such as *DCN* and *SFRP4*, and 214 genes upregulated in cancer cells, such as *SPRR1B* and *SLURP2* (Supplementary Fig. 5a). We then performed pathway enrichment of these dysregulated genes. Cell proliferation-related functions were more enriched in tumor-derived fibroblasts, such as “epithelial cell migration” and “response to fibroblast proliferation”, whereas cell communication-related functions showed high enrichment in cancer cells, such as “cell-cell junction assembly” and “positive regulation of leukocyte cell-cell adhesion” (Supplementary Fig. 5b). These results indicated that fibroblasts from tumor promoted the tumor progression.

### CD8^+^ T cells showed high diversity and developed exhausted intra-tumoral subtypes in CC

**Fig. 4:**
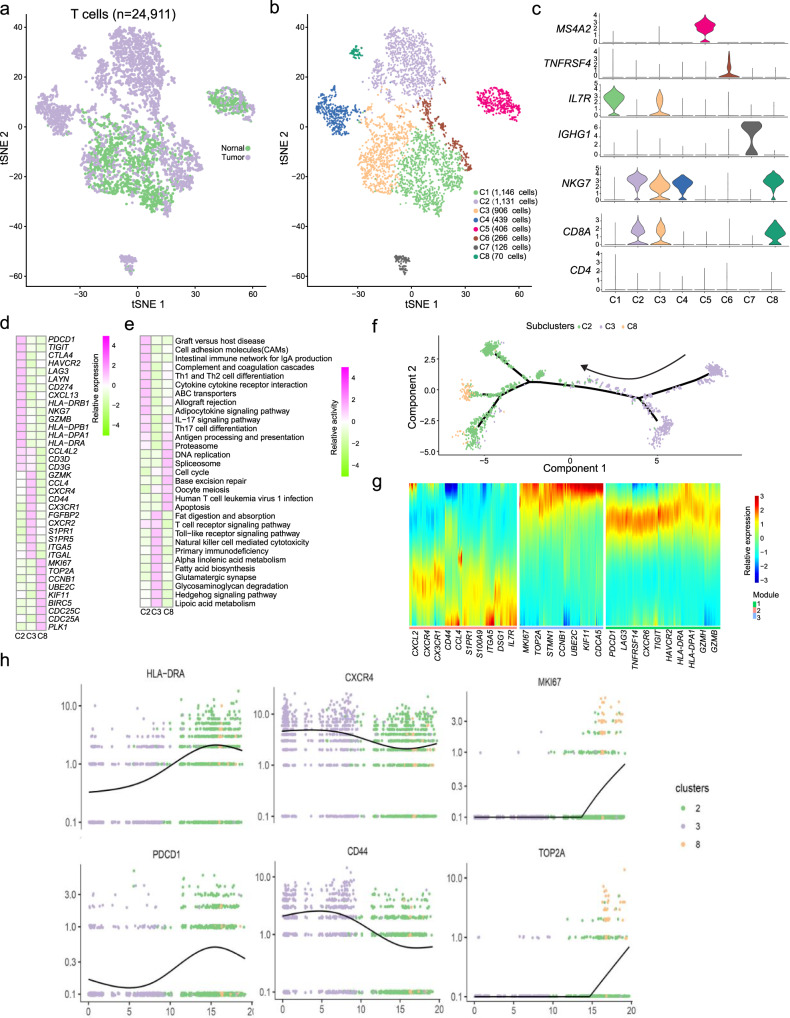
The heterogeneity of CD8^+^ T cells in cervical cancer samples. **a** tSNE plot showing CD8^+^ T cells in tumor and NAT cervical samples. **b** tSNE plot showing subclusters of CD8^+^ T cells. **c** The relative expression levels of the *CD4*, *CD8A*, *NKG7*, *IGHG1*, *IL7R*, *TNFRSF4*, and *MS4A2* gene across 8 subclusters of CD8^+^ T cells. **d** Heatmap showing the relative expression of top marker genes in the C2, C3, and C8 subcluster. **e** Heatmap showing the relative activities of significant biological processes in the C2, C3, and C8 subcluster. **f** Pseudo-time trajectory of the C2, C3, and C8 subcluster. **g** The expression patterns of genes in the identified modules. **h** The expression levels of the gene *HLA-DRA*, *CXCR4*, *MKI67*, *PDCD1*, *CD44*, and *TOP2A* along the pseudo-time trajectory across C2, C3 and C8 subclusters.

With 24,911 cells detected, T cells represented the most prevalent cell type in our scRNA-seq data (Fig. 4a). Our re-clustering analysis revealed eight T cell subclusters, which were designated as CD8^+^ T cells (*CD8A*^+^, cluster C2, C3 and C8), natural killer T cells (*NKG7*^+^, cluster C4) and memory T cell (*IL7R*^+^, cluster C1), plasma cells (*IGHG1*^+^, cluster C7), regulatory T cells (*TNFRSF4*^+^, cluster C6) and mast cell (*TPSB2*^+^, cluster C5) (Fig. 4b, c, Supplementary Data 4). We observed that CD8^+^ T cells highly expressed CD8^+^ T cell markers, but they had almost no expression of the CD4^+^ T cell markers. Then, we analyzed the difference between CD8^+^ T cell clusters, which represented a large proportion of CD8^+^ T cells in both tumor and normal tissues. The C3 cluster CD8^+^ T cells (*CXCR4*^+^) were characterized by the high expression of the *GZMK*, *CXCR2* and *CX3CR1* gene, commonly associated with effective T cells and the low expression of check point genes (*PDCD1*, *TIGIT*, *CTLA4*, *HAVCR2*, *LAG3* and *CD274*), suggesting that these cells are precursors of cytotoxic T cells (Fig. 4d, Supplementary Data 4). In addition, our analysis also revealed the high expression of some CD8^+^ T cell migration regulators, such as chemokine receptors (*CX3CR1*, *CXCR4*, and *CXCR2*), S1P receptors (*S1PR1* and *S1PR5*), and integrins (*ITGA5* and *ITGAL*). High expressed genes of the C3 cluster CD8^+^ T cells (*CXCR4*^+^) were found to be enriched with such pathways as NK cells medicated cytotoxicity, T cells receptor signaling and Toll-like receptor signaling pathway, which was related to the cytotoxic function (Fig. 4e).

The C2 cluster CD8^+^ T cells (*PDCD1*^+^) were characterized by the high expression of immune checkpoint genes (such as *PDCD1*, *TIGIT*, *CTLA4*, *HAVCR2*, *LAG3*, and *CD274*). Meanwhile, the DEG analysis also revealed higher expression of *HLA-DPA1*, *HLA-DRA*, and *HLA-DRB1*, which is beneficial to the antigen presentation and the activation of cytotoxicity T cells. Functional analysis revealed the enrichment of the immune-related pathways, such as cell-adhesion molecular, ABC-transports, Th17 cell differentiation, and complement cascades. The C8 cluster CD8^+^ T cells (*MKI67*^+^) presented proliferative cells as they expressed high levels of gene *MKI67*, *TOP2A*, and *CCNB1*, and low levels of gene *TIGIT*, *CTLA4*, *PDCD1*, *HAVCR2*, *LAG3*, and *LAYN*. Functional enrichment analysis revealed that these cells were enriched with pathways related to cell proliferation (cell cycle, oocyte-meiosis, DNA replication and base excision repair), suggesting limited effective ability of these cells.

To further investigate how different CD8^+^ T cell subtypes developed in CC, we performed pseudo-time trajectory analysis of all CD8^+^ T cells (see “Methods”). Our analysis showed that the C3 cluster CD8^+^ T cells (*CXCR4*^+^) cells were at the beginning of the trajectory path, whereas the C2 cluster CD8^+^ T cells (*PDCD1*^+^) and the C8 cluster CD8^+^ T cells (*MKI67*^+^) were at a terminal state (Fig. 4f). This was accompanied by the increased expression of exhaustion markers *PDCD1*, *LAG3*, and *TIM3*, and the decrease of effector markers, such as *CX3CR1*, *CXCR4*, and *CXCR2* (Fig. 4g). Meanwhile, we found that *MKI67*, *TOP2A* and *CCNB1* increased at one end of the pseudo-temporal trajectory (Fig. 4h), and demonstrated that cell populations with both proliferative and exhausted states were present. The observation suggested that some cells might be reserved to have proliferative ability before being terminally exhausted.

### Deconvolution of bulk RNA-seq data revealed four different CC subtypes

**Fig. 5:**
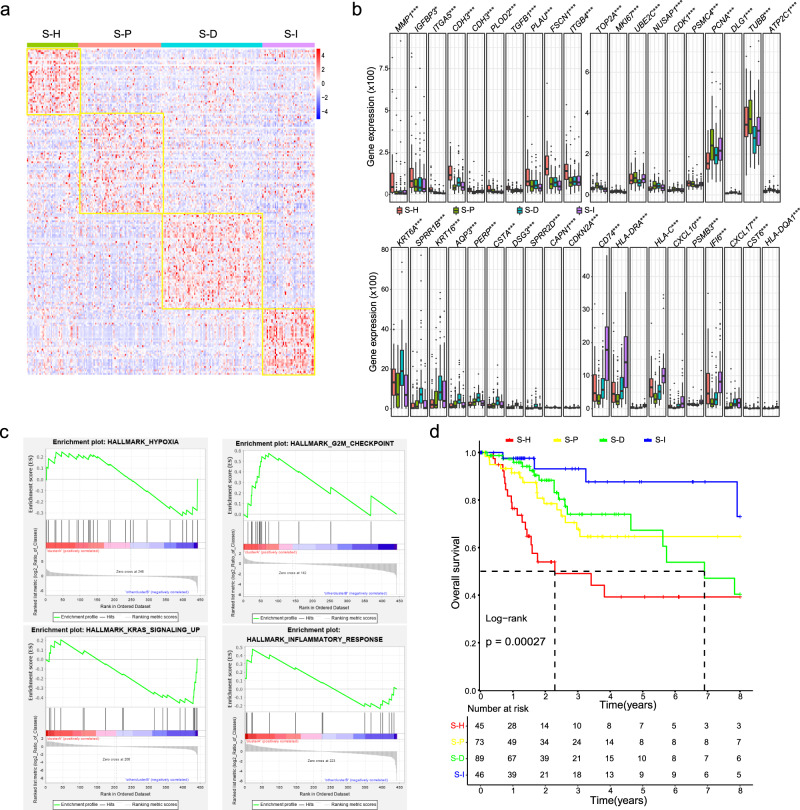
Differences among the four CC subtypes. **a** Subclusters of CC samples based on signature genes from scRNA-seq data. **b** Bar plots showing the comparisons of expression levels of representative subcluster markers genes across different subclusters. **c** GSEA enrichment of signature genes in each CC subtype. **d** Survival differences among the four subclusters in CC samples. *P < 0.05, *P < 0.01, ***P < 0.001.

Our single-cell transcriptomics analysis revealed highly different subpopulations in many cell types, suggesting a more precision heterogenous property of CC. In CC, three subtypes have been identified largely based on mRNA expression of certain genes, including two squamous subtypes (Keratin-high and Keratin-low), and an adenocarcinoma-rich subtype (adenocarcinoma). However, these subtypes did not reflect the heterogenous TME compositions and the prognostic differences. We further classified CC subtypes by using cell type-specific genes that were highly expressed by the malignant cells. Our classification strategy reduced the effect of non-malignant cells in CC. We used the 705 marker genes of epithelial cells between normal and tumor tissues (log FC > 1.5 and *P* < 0.05) from scRNA-seq data and divided 253 samples of the TCGA CESC cohort into four major subtypes (Fig. 5a), namely the hypoxia (S-H subtype), proliferation (S-P subtype), differentiation (S-D subtype), and immunoactive (S-I subtype) subtype. The S-H subtype expressed 10 significant genes (*MMP1*, *IGFBP3*, *ITGA5*, *CDH3*, *ICAM1*, *FLOD2*, *TGFB1*, *PLAU*, *FSCN1*, and *ITGB4*) (Fig. 5b), and presented the enrichment of hypoxia (Fig. 5c). Hypoxia is one of most common tumor characteristics, which is mainly caused by insufficient vascularization^16^. The hypoxic TME condition impedes immune response by recruiting immunosuppressive cells and genes. The S-P subtype expressed 10 significant genes (*TUBB*, *UBE2C*, *NUSAP1*, *CKD1*, *PSMC4*, *PCNA*, *DLG1*, *ATP2C1*, *MKI67*, and *TOP2A*) and presented enrichment of cell proliferation. The S-D subtype expressed 10 significant genes (*KRT6A*, *SPRR1B*, *KRT16*, *AQP3*, *PERP*, *CSTA*, *DSG3*, *SPRR2D*, *CAPN1* and *CDKN2A*) that showed enrichment of cell differentiation. The S-I subtype expressed 10 significant genes (*HLA-DMA*, *CD74*, *HLA-DMA*, *HLA-C*, *CXCL10*, *PSMB3*, *IFI6*, *CXCL17*, *CST6* and *HLA-DQA1*), and presented enrichment of immune-related biological processes. Furthermore, we obtained gene expression profiles of 340 cervical cancer samples from the GEO database (GSE15166, GSE29617, and GSE68335) and divided them into four subtypes using the same gene marker set (Supplementary Fig. 6a). These subtypes expressed similar signature genes and biological processes (Supplementary Fig. 6b–f). Then, we compared the prognosis of different subtypes. These four subtypes showed significantly different overall survival times (Fig. 5d). The S-I showed the longest survival time, while the S-H subtype exhibited the worst prognosis. To further explore the differences between the four CC subtypes, we performed GSVA analysis to evaluate scores of 50 hallmarks in each sample (see Supplementary methods). Different subtypes were specifically enriched in different hallmarks (Supplementary Fig. 7a). In particular, the S-D subtype was specifically enriched in “MYC targets v1” and “protein secretion”-related hallmark gene sets. The S-P subtype was highly enriched in “Wnt β catenin signaling”, “HEME metabolism” and “myogenesis”-related hallmark gene sets. The S-I subtype showed high scores of “IL6 JAK STAT3 signaling”, “oxidative phosphorylation”, and “IL2 STAT5 signaling”-related hallmark gene sets. The S-H subtype specifically high enrichment of “reactive oxygen species pathway” and “hypoxia”-related hallmarks. We also performed CIBERSORT analysis to infer the relative abundance of immune cells in each sample (see Supplementary methods). The four CC subtypes showed distinct infiltration of different immune cell types (Supplementary Fig. 7b). For example, the S-I subtype showed higher infiltration of CD8^+^ T cells and regulatory T cells. In addition, we compared the expression levels of immune checkpoint genes between different CC subtypes. The S-I subtype showed significantly high expression of many immune checkpoints, such as *BTLA*, *CD27*, and *TIGIT* (Supplementary Fig. 7c).

## Discussion

In this study, we employed scRNA-seq to comprehensively delineate the cellular heterogeneity of human CCs. By using the signature genes derived from scRNA-seq data analysis, we identified four molecular subtypes of CCs, namely the hypoxia, proliferative, differentiated, and immunoactive subtype. The stratification of CC tumors not only promotes our understanding of its etiologies, but also accelerates the development of personalized therapeutic strategies for CC patients.

We calculated the percentages of the subpopulations of epithelial cells, fibroblasts, and T cells in CC tumor and paired NAT samples (Supplementary Fig. 8). Some cell subpopulations showed acceptable variations in different tumor or NAT samples, such as the C1, C3, and C5 T cells in NAT samples, while some showed large variations in different tumor or NAT samples, such as the C1, C2, and C3 epithelial cells in tumor samples. The large percentage variations of some cell subpopulations might be due to heterogeneity between different patients and sample collections. Cell subpopulations are cells with specific status under specific conditions, some of which showed great heterogeneity between different patients^17–19^. Samples used for scRNA-seq are randomly chosen from pathological or related areas, but cells are not uniformly distributed. Increasing the number of samples could, to some extent, reduce the bias induced by these limitations, but also enlarges the volume of cell subpopulations. Cell subpopulations identified from limited number of samples might show large variations between samples, but reflect the existence of specific cell status.

One significant advantage of our CC classification was that our strategy was based on the epithelial cell markers from scRNA-seq data. The strategy could eliminate the influence of other cells such as, stromal and immune cell markers in the categorizing process. For example, in early phase, HGS-OvCa was identified as four molecular subtypes: immunoreactive, differentiated, proliferative and mesenchymal according to the TCGA data. However, a recent study from scRNA-seq data found that the HGSOC classification of immunoreactive and mesenchymal reflected the infiltration of immune cells and fibroblasts, but ignored malignant cells. The scRNA-seq data had the advantage over the bulk RNA-seq in focusing on the tumor cells. Another advantage is that the scRNA-seq data could help understand the potential mechanism of tumor progression. EMT plays key roles in the development and pathological biology of tumor, and understanding its regulation is important for developing new therapeutic intervention for tumor patients^20^. In addition, emerging evidence has shown that hypoxia could affect EMT by regulating the expression of EMT-related transcription factors and signaling genes^21,22^.

Accumulating evidence has demonstrated that immune cells in TME, such as tumor-associated macrophages and T cells, are closely involved in the progression of tumor. The TME, including immune cells with malleable states and their communications with other cells, is a major contributor to regulating immune response against tumor cell behaviors^23^. To our best knowledge, our study presented the first single-cell landscape of infiltrating immune cells in CC. We observed that CD8^+^ T cells were infiltrating with different status in CC samples, including proliferative and exhausted status, and activated CD8^+^ T cells were in low abundance. In the TME, CD8^+^ T cells are the major effector to kill tumor cells^24^. The immunosuppressed state of CD8^+^ T cells indicated the lack of sufficient activated T cells to kill tumor cells in the TME of CC. We found that both inhibitory receptors (IRs) and activation markers of T cell exhaustion were expressed in some CD8^+^ T cells. Whether these CD8^+^ T cells turn into effective or exhausted state was determined by the expression modulation of IRs. We further revealed the differentiation trajectory of different CD8^+^ T cells in CC wherein *CX3CR1*^+^ CD8^+^ T cells transformed to the *PDCD1*^+^ CD8^+^ T cells.

We found that different subtypes presented various infiltration of immune cells, especially for CD8^+^ T cells. CC patients of the immunoactive subtype might respond to immune checkpoint blockade (ICB) therapy, but patients of other subtypes may not. Recently, novel ICB targets beyond CTLA4 and PD-1 have been identified, such as LAG3, TIM3, HAVCR2, and TIGIT^25^. Numerous clinical trials of these emerging ICB targets are underway. Overall, CD8^+^ T cells showed high expression level of LAG3 and TIM3 in our scRNA-seq data. Our analysis suggest that LAG3 and TIM3 might be potential ICB targets that are worth further investigation for the ICB therapy of CC patients.

In conclusion, our study characterized the single-cell landscape of TME in CC. Then, we firstly classified all CESCs patients into four subtypes, which may present different response to immune checkpoint inhibitors. Although more datasets and experimental validation are needed, our results shed lights on T cell infiltration and response in CC, which might promote the development of more personalized diagnostic and therapeutic strategies in clinical practice.

## Methods

### Clinical specimen collection

Human cervical samples were collected from three different patients in the Obstetrics and Gynecology Hospital of Fudan University, including three cancer samples and paired adjacent non-tumor (NAT) samples. All patients gave informed consent. The clinical information, including age, menstrual status, FIGO stage, histological type, HPV status, and treatment, are provided in Supplementary Table 1. The estrogen hormone of all included patients was at low levels. This study was approved and supervised by the ethics committee of the Obstetrics and Gynecology Hospital of Fudan University.

### Sample preparation and single-cell isolation

Collected fresh cervical samples were washed with 1× PBS three times. Then tissue samples were cut into 1 mm^3^ pieces and incubated in the same dispase solution at 37 °C for half an hour. Pieced tissue was gently dissociated with a pipette and incubated in trypsin 0.05% solution diluted with PBS for 10 min. Single-cell samples were filtered out with a 70 mm filter after the trypsin was deactivated by RPMI 1640 medium (Gibco), supplemented with 10% FBS and 1% penicillin/streptomycin (Invitrogen). The trypan blue microscopy was used to determine the percentage of active cells, and only samples with no less than 85% of active cells were used for scRNA-seq. Single cells were then counted with a hemocytometer and live cells were sorted for the preparation of 10X Genomics scRNA-seq library.

### Single-cell RNA sequencing

The single-cell suspension was loaded onto a 10X Chromium Single-Cell instrument to generate single-cell Gel Beads-in-emulsion (GEMs). The single-cell RNA library was then constructed and estimated by using 10X Genomics Chromium Single-cell 30 Library, Gel Bead & Multiplex Kit. The scRNA-seq was performed on the Illumina NextSeq500. All procedures were performed according to the standard manufacturer’s protocol.

### ScRNA-seq data processing

The raw scRNA-seq reads were first processed for sample demultiplexing, barcode processing, and genome mapping by using the Cell Ranger (version 3.0.1)^26^ software. The GRCh38 human reference genome was utilized in the read alignment process. The unique molecular identifiers (UMIs) were counted in each single cell. Low-quality cells were filtered as previously described^12^. Specifically, cells with UMI number <200, gene number <200, or percentage of mitochondrion-derived UMI counts >10% were discarded as low-quality cells. The Seurat R package (version 4.0)^27^ was applied in the quality control procedure. In addition, the Scrublet software (version 0.2.2)^28^ was employed to identify and remove potential doublets. After removing low-quality and doublet cells, data of all samples was normalized and merged. The feature expression measurements for each cell were normalized by the total expression by using the “LogNormalize” method implemented in the NormalizeData function. Then normalized counts were then multiplied by a scale factor (10,000) and log-transformed.

### Dimension reduction and unsupervised clustering

The normalized data was used to identify gene features with high cell-to-cell variations by utilizing the FindVariableFeatures function. The top 2000 highly variable genes were used to scale the data by using the ScaleData function. Then the principal component analysis (PCA) was adopted to reduce data dimensions. The FindNeighbors and FindClusters functions were consecutively used to perform a graph-based clustering and find the optimal cluster resolution. The RunTSNE function was applied for appropriate visualization. Differentially expressed gene markers in each cluster were identified by the FindAllMarkers function, which compares gene expression with those in all other cell clusters.

### Cell type annotation

The unbiased cell type recognition was performed by applying the SingleR package (version 1.4.1)^29^, which leverages reference transcriptomic datasets of pure cell types. Then the annotated cell clusters were checked by manually curated gene markers retrieved from the CellMarker database^30^ and published papers^31,32^. The differential genes were then identified in each cell type with the following criteria: expressed in at least 20% of cells in either sample groups; |log_2_FoldChange| >0.585; adjusted p value < 0.01.

### Copy number alteration inference

The inferCNV software (https://github.com/broadinstitute/infercnv) was applied to infer copy number alterations (CNAs) in our scRNA-seq data. CNAs were computed according to a previous study^33^. Briefly, genes were sorted by their chromosomal locations to evaluate initial CNAs from expression levels. A sliding window of 100 genes was used to calculate moving averages of relative expression values in each chromosome. In each epithelial cell, the relative CNAs were calculated from the inferCNV outputs. For each bin of 30 genes, an average value of CNA was estimated in nonoverlapping genomic regions. Average CNA values were rounded to the closest integers.

### Pseudo-time trajectory analysis

The pseudo-time trajectory was inferred by utilizing the Monocle2 package (version 2.8.0)^34^ to reveal the cell-state transitions. The following parameters were adopted: average expression R0.125, num_cells_expressed R10, qval < 0.01 (differentialGeneTest function). The DDRTree function was applied to reduce the dimensions with default settings. The expression and variance levels were used to determine the ordering genes.

### Functional enrichment analysis

Functional enrichment analyses in this study were conducted using the clusterProfiler R package (version 4.1)^35^. Differential genes in each cell type or cluster were used to compute enriched GO biological processes or KEGG pathways. The GSEA analysis was performed by using the gsea function. GO terms or KEGG pathways with adjusted *p* value < 0.05 were considered as significantly enriched by the gene sets of interest.

### Gene regulatory network analysis

The Single-Cell rEgulatory Network Inference and Clustering (SCENIC) method was employed to perform gene regulatory network analysis in different cell types or clusters. The SCENIC analysis was realized by the pySCENIC (version 0.10.2) software^36^. Briefly, the processing consists of three major steps^37^. First, co-expression modules of transcription factors (TFs) and targets were inferred by using the gradient boosting machine regression implemented in GRNBoost2^38^. Second, these modules were optimized to remove indirect targets by using the i-cisTarget software^39^. Third, enrichment scores for the regulons’ targets were calculated by the AUCell algorithm^36^ to estimate the activity of these regulons. The TFs and target motifs were collected by the SCENIC group.

### Statistics and reproducibility

Statistical analysis and data visualization in the present study was performed by using the R software (version 4.0.2, R Foundation for Statistical Computing, Vienna, Austria; http://www.r-project.org). Unless specifically stated, *p* or FDR values < 0.05 were considered as statistically significant.

### Reporting summary

Further information on research design is available in the Nature Portfolio Reporting Summary linked to this article.

## Supplementary information

Supplementary Information

Description of Additional Supplementary Files

Supplementary Data 1

Supplementary Data 2

Supplementary Data 3

Supplementary Data 4

Reporting Summary

## Data Availability

Single-cell RNA sequencing gene expression data generated in this study has been deposited in the ArrayExpress database with accession of E-MTAB-11948. Any other data are available from the corresponding author on reasonable request. Software and resources used for analysis and plotting are described in each method section.

## Code availability

The R scripts used for analysis and visualization are available upon reasonable request to the corresponding author.

## References

[1] Siegel RL, Miller KD, Jemal A (2020). Cancer statistics, 2020. CA Cancer J. Clin..

[2] Sung H (2021). Global cancer statistics 2020: GLOBOCAN estimates of incidence and mortality worldwide for 36 cancers in 185 countries. CA Cancer J. Clin..

[3] Schiffman M (2011). Human papillomavirus testing in the prevention of cervical cancer. J. Natl Cancer Inst..

[4] Moody CA, Laimins LA (2010). Human papillomavirus oncoproteins: pathways to transformation. Nat. Rev. Cancer.

[5] Stryker ZI, Rajabi M, Davis PJ, Mousa SA (2019). Evaluation of angiogenesis assays. Biomedicines.

[6] Minion LE, Tewari KS (2018). Cervical cancer - state of the science: from angiogenesis blockade to checkpoint inhibition. Gynecol. Oncol..

[7] Cancer Genome Atlas Research N (2017). Integrated genomic and molecular characterization of cervical cancer. Nature.

[8] Zhu X (2022). Subtyping of human papillomavirus-positive cervical cancers based on the expression profiles of 50 genes. Front. Immunol..

[9] Wang X, Xu C, Sun H (2022). DNA damage repair-related genes signature for immune infiltration and outcome in cervical cancer. Front. Genet..

[10] de Geus V (2021). Identifying molecular changes in early cervical cancer samples of patients that developed metastasis. Front. Oncol..

[11] Wang S (2022). SERPINB3 (SCCA1) inhibits cathepsin L and lysoptosis, protecting cervical cancer cells from chemoradiation. Commun. Biol..

[12] Li C, Guo L, Li S, Hua K (2021). Single-cell transcriptomics reveals the landscape of intra-tumoral heterogeneity and transcriptional activities of ECs in CC. Mol. Ther. Nucleic Acids.

[13] Zhang Y, Liu Z (2017). STAT1 in cancer: friend or foe?. Discov. Med..

[14] Adamkova L, Souckova K, Kovarik J (2007). Transcription protein STAT1: biology and relation to cancer. Folia Biol..

[15] Zhang X, Li X, Tan F, Yu N, Pei H (2017). STAT1 inhibits MiR-181a expression to suppress colorectal cancer cell proliferation through PTEN/Akt. J. Cell. Biochem..

[16] Hanahan D (2022). Hallmarks of cancer: new dimensions. Cancer Discov..

[17] Hu Z (2020). The repertoire of serous ovarian cancer non-genetic heterogeneity revealed by single-cell sequencing of normal fallopian tube epithelial cells. Cancer Cell.

[18] Ma L (2019). Tumor cell biodiversity drives microenvironmental reprogramming in liver cancer. Cancer Cell.

[19] Jackson HW (2020). The single-cell pathology landscape of breast cancer. Nature.

[20] Brabletz T, Kalluri R, Nieto MA, Weinberg RA (2018). EMT in cancer. Nat. Rev. Cancer.

[21] Hapke RY, Haake SM (2020). Hypoxia-induced epithelial to mesenchymal transition in cancer. Cancer Lett..

[22] Chen XJ (2019). Hypoxia-induced ZEB1 promotes cervical cancer progression via CCL8-dependent tumour-associated macrophage recruitment. Cell Death Dis..

[23] Zhao J (2020). Single cell RNA-seq reveals the landscape of tumor and infiltrating immune cells in nasopharyngeal carcinoma. Cancer Lett..

[24] Liu Y (2021). Tumour heterogeneity and intercellular networks of nasopharyngeal carcinoma at single cell resolution. Nat. Commun..

[25] Qin S (2019). Novel immune checkpoint targets: moving beyond PD-1 and CTLA-4. Mol. Cancer.

[26] Zheng GX (2017). Massively parallel digital transcriptional profiling of single cells. Nat. Commun..

[27] Hao Y (2021). Integrated analysis of multimodal single-cell data. Cell.

[28] Wolock SL, Lopez R, Klein AM (2019). Scrublet: computational identification of cell doublets in single-cell transcriptomic data. Cell Syst..

[29] Aran D (2019). Reference-based analysis of lung single-cell sequencing reveals a transitional profibrotic macrophage. Nat. Immunol..

[30] Zhang X (2019). CellMarker: a manually curated resource of cell markers in human and mouse. Nucleic Acids Res..

[31] Yao T (2015). Cervical cancer stem cells. Cell Prolif..

[32] Patterson AL (2020). Putative human myometrial and fibroid stem-like cells have mesenchymal stem cell and endometrial stromal cell properties. Hum. Reprod..

[33] Wang R (2021). Single-cell dissection of intratumoral heterogeneity and lineage diversity in metastatic gastric adenocarcinoma. Nat. Med..

[34] Trapnell C (2014). The dynamics and regulators of cell fate decisions are revealed by pseudotemporal ordering of single cells. Nat. Biotechnol..

[35] Yu G, Wang LG, Han Y, He QY (2012). clusterProfiler: an R package for comparing biological themes among gene clusters. OMICS.

[36] Aibar S (2017). SCENIC: single-cell regulatory network inference and clustering. Nat. Methods.

[37] Van de Sande B (2020). A scalable SCENIC workflow for single-cell gene regulatory network analysis. Nat. Protoc..

[38] Moerman T (2019). GRNBoost2 and Arboreto: efficient and scalable inference of gene regulatory networks. Bioinformatics.

[39] Imrichova H, Hulselmans G, Atak ZK, Potier D, Aerts S (2015). i-cisTarget 2015 update: generalized cis-regulatory enrichment analysis in human, mouse and fly. Nucleic Acids Res..

