# Supplementary material for: Single-cell transcriptomics reveals cellular heterogeneity and molecular stratification of cervical cancer
Source: Commun Biol. 2022 Nov 10;5:1208. doi: 10.1038/s42003-022-04142-w (PMC9649750; doi:10.1038/s42003-022-04142-w)
Supplement: Supplementary file 1 — Supplementary Information [file 42003_2022_4142_MOESM1_ESM.pdf]

Supplementary Tables

Supplementary Table 1. Clinical information of CC patients.

Patients	Age (year)	Menstrual status	FIGO stage	Histological type	HPV status	Treatment
P1	48	menstruating	IB1	Squamous cell carcinoma	HPV16(+)	Radical hysterectomy
P2	50	menopause	IB1	Squamous cell carcinoma	HPV16, 33(+)	Radical hysterectomy
P3	51	menopause	IB1	Squamous cell carcinoma	HPV16(+)	Radical hysterectomy

Supplementary Table 2. Expression of top gene markers in epithelial subclusters.

Gene	C1	C2	C3	C4	C5
MMP1	1.64	0.0082	0.040	0.99	0.047
SPRR1B	1.91	0.0083	0.0042	0.95	0.0091
PI3	2.96	0.063	0.044	2.53	0.28
KRT16	2.26	0.17	0.14	1.51	0.24
S100A9	4.14	0.88	0.54	3.34	1.10
SLURP2	3.00	0.15	0.11	2.84	0.079
S100A8	3.75	0.20	0.10	2.94	0.43
ERO1A	1.51	0.51	0.36	0.89	0.36
LY6D	4.40	0.78	0.47	4.42	0.26
SERPINB13	2.40	0.47	0.32	2.09	0.0070
GJB2	1.57	0.23	0.14	0.90	0.13
SERPINB3	3.35	1.32	0.68	3.12	0.23
CSTB	3.20	1.99	1.24	2.70	1.56
KRT6B	1.10	0.092	0.041	0.68	0.054
MMP10	0.67	0.028	0.021	0.34	0.051
CSTA	2.46	1.24	0.79	1.93	0.023
ECM1	1.04	0.11	0.11	0.58	0.0071
DSG3	2.07	0.33	0.27	1.71	0.019
GJB6	1.06	0.0087	0.0047	0.50	0.011
KRT6A	3.65	1.70	1.18	3.38	0.15
HLA-DQA1	0.0080	3.05	2.12	0.0071	0.24
HLA-DRB5	0.034	3.14	2.20	0.047	0.26
HLA-DPB1	0.19	2.84	1.81	0.25	0.86
HLA-DRA	1.31	4.00	2.78	1.51	1.84
HLA-DPA1	0.42	2.84	1.74	0.51	1.01
RARRES1	0.10	2.47	1.42	0.083	1.51
CP	0.16	2.40	1.50	0.15	1.21
HLA-DRB1	0.97	3.33	2.36	1.14	1.84
IL32	1.10	3.17	2.47	1.17	0.14
CD74	1.28	3.89	3.40	1.47	2.44
HSPD1	0.65	3.10	2.85	0.94	1.03
HLA-A	2.91	4.50	3.78	3.05	1.72
ZFAND2A	0.30	1.86	1.43	0.37	0.19
HLA-F	0.63	2.27	1.70	0.82	0.35
IGFBP7	0.30	2.07	1.47	0.34	0.30
HLA-B	2.86	4.21	3.39	3.09	2.20
HLA-G	0.0018	1.29	0.83	0.0012	0.0040
TNFRSF12A	0.54	1.76	1.21	0.49	0.74
HLA-DQB1	0.14	1.61	1.23	0.18	0.58
HLA-DMA	0.31	1.72	1.02	0.46	0.87
CCDC80	0.12	0.20	0.70	0.10	0.11
IER5	0.51	1.23	1.91	0.58	0.51

MAFB	0.053	0.17	0.80	0.043	0.38
HIST1H4C	0.072	0.18	1.05	0.37	0.80
MKI67	0.078	0.10	1.02	0.95	0.0081
SOX4	0.47	1.16	1.60	0.42	1.13
GADD45B	0.20	0.60	1.46	0.23	1.78
HEXIM1	0.17	0.23	0.86	0.23	0.80
HMGB2	0.27	0.77	1.47	1.26	0.27
UBE2S	0.28	0.68	1.20	1.30	0.57
HSPH1	0.29	2.62	2.62	0.33	0.69
MALAT1	5.30	5.44	6.36	4.97	5.83
MUC16	0.32	0.36	0.85	0.21	0.79
TLE4	0.28	0.44	0.99	0.21	0.44
PARD6G-AS1	0.050	0.28	0.66	0.047	0.055
HSPA6	0.27	1.13	1.39	0.27	0.10
TRA2B	0.85	1.09	1.71	0.94	0.83
COL7A1	0.51	0.35	0.99	0.58	0.013
PLK2	0.11	0.76	1.10	0.095	0.66
UBE2C	0.060	0.089	0.61	1.10	0.0060
TOP2A	0.064	0.078	0.70	1.20	0.012
ANLN	0.10	0.13	0.46	1.08	0.012
CDK1	0.19	0.089	0.58	1.08	0.0048
BIRC5	0.041	0.019	0.16	0.88	0.0046
CDKN3	0.058	0.091	0.46	0.89	0.0064
ASPM	0.030	0.035	0.48	0.83	0.0049
KPNA2	0.19	0.24	0.63	0.96	0.25
CDC20	0.033	0.059	0.30	0.72	0.019
CEP55	0.061	0.032	0.27	0.77	0.0017
ARL6IP1	0.36	0.52	0.87	1.30	0.84
H2AFX	0.18	0.15	0.51	1.01	0.32
CCNB2	0.046	0.048	0.28	0.72	0.0036
CENPF	0.046	0.13	0.79	0.94	0.018
CCNB1	0.049	0.060	0.42	0.70	0.049
SMC4	0.43	0.64	1.08	1.32	0.12
DLGAP5	0.017	0.013	0.30	0.63	0.0013
SCGB3A1	0.020	0.042	0.026	0.015	4.20
TFF3	0.0053	0.00019	0.00	0.011	2.97
BPIFB1	0.00011	0.0046	0.0017	0.00047	2.37
PIGR	0.00068	0.21	0.10	0.0018	3.16
SCGB2A1	0.012	0.014	0.0098	0.0090	2.16
MUC5B	0.00020	0.0021	0.0093	0.00070	2.87
SAA1	0.21	0.22	0.13	0.27	2.74
LCN2	0.73	0.26	0.13	0.59	3.21
WFDC2	0.85	3.00	2.04	0.77	5.19

CXCL2	0.020	0.47	0.65	0.028	2.61
CXCL3	0.022	0.42	0.50	0.027	2.36
AGR2	0.38	0.63	0.29	0.45	3.24
SLPI	2.80	2.79	1.87	2.49	5.07
SCGB1D2	0.00	0.0017	0.0027	0.00	0.89
FCGBP	0.0018	0.0018	0.0061	0.0014	1.61
LTF	0.011	0.0080	0.0045	0.013	1.86
CXCL1	0.20	0.78	0.71	0.19	2.32
CLU	0.40	0.58	0.69	0.36	2.98
SAA2	0.025	0.028	0.025	0.022	1.48

Supplementary Table 3. Relative expression of differential transcription factors in each cancer epithelial cell subcluster.

Regulon	C1	C2	C3	C4	C5
HIF1A (93g)	1.89	-0.40	0.59	-0.098	-0.037
TFDP1 (98g)	1.84	-0.62	-0.34	0.99	-0.62
GRHL1 (103g)	1.62	-0.59	-0.56	0.43	-1.11
IRF9 (45g)	1.41	-0.17	-0.17	1.34	-0.40
STAT2 (86g)	1.41	-0.34	-0.088	1.37	-0.45
TP63 (87g)	1.36	-1.08	-0.82	0.71	-1.08
GRHL3 (27g)	1.36	-0.95	-0.95	0.68	-0.95
IRF7 (43g)	1.34	-0.051	-0.13	1.37	-0.51
DDIT3 (10g)	1.20	0.28	0.24	1.23	-0.98
YY1 (60g)	0.52	-1.95	0.63	0.45	-0.44
IRF6 (74g)	0.99	0.43	0.14	-0.098	-2.08
CEBPB (17g)	0.90	-1.49	-0.54	-0.60	1.50
BCL3 (14g)	0.51	-0.50	0.28	-0.68	1.93
XBP1 (680g)	-0.26	-0.50	-0.28	-0.42	2.26
HOXB6 (11g)	-0.38	-0.38	-0.38	-0.38	2.27
HIVEP2 (12g)	-0.38	-0.38	-0.38	-0.38	2.27
SPDEF (25g)	-0.54	0.066	-0.10	-0.54	2.20
ATF4 (24g)	-0.60	-1.08	-0.85	-0.11	0.46
REL (97g)	-0.65	-0.17	0.45	-0.86	2.05
NFKB1 (196g)	-0.49	0.48	1.29	-1.10	1.25
E2F7 (126g)	-0.67	-0.065	2.01	0.58	-0.67
STAT3 (20g)	-0.68	0.14	1.94	-0.68	0.62
FOXC1 (12g)	-0.88	0.35	1.85	-0.88	0.34
IRF1 (76g)	-0.90	0.57	1.74	-1.12	0.44
RFX3 (92g)	-0.41	-0.19	2.26	-0.41	-0.41
BRCA1 (61g)	-0.43	-0.43	2.25	-0.073	-0.43
STAT1 (936g)	0.048	1.32	1.31	0.057	-0.70
FOSL1 (363g)	-0.70	1.53	1.33	-0.91	-0.42

Supplementary Figures

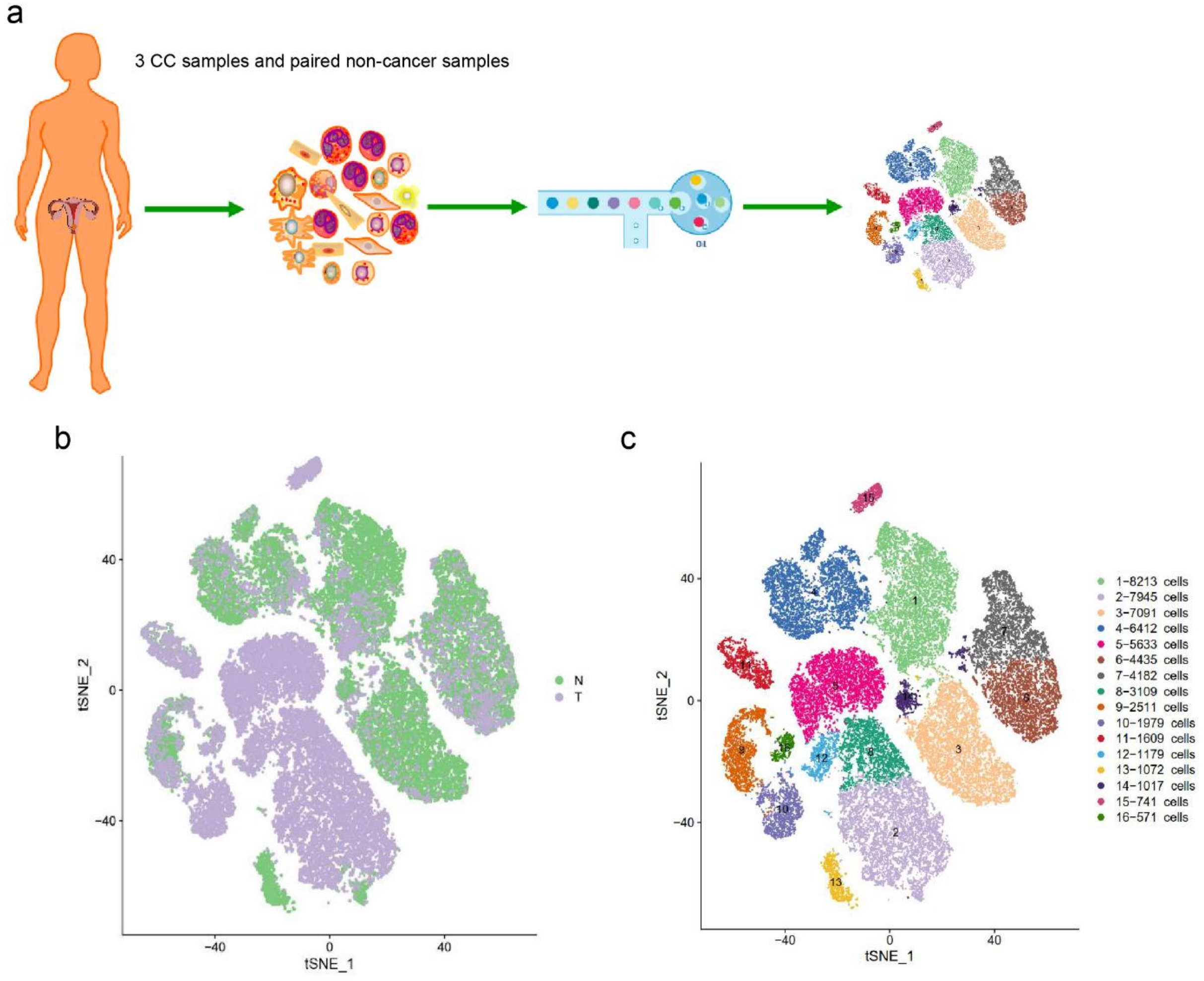

Supplementary Fig. 1 ScRNA-seq of CC tumor and paired non-tumor samples. a Schematic of overall design for this study. **b** tSNE plot showing cells in CC tumor (T) and non-

tumor (N) samples. **c** tSNE plot showing 16 different cell clusters in all samples.

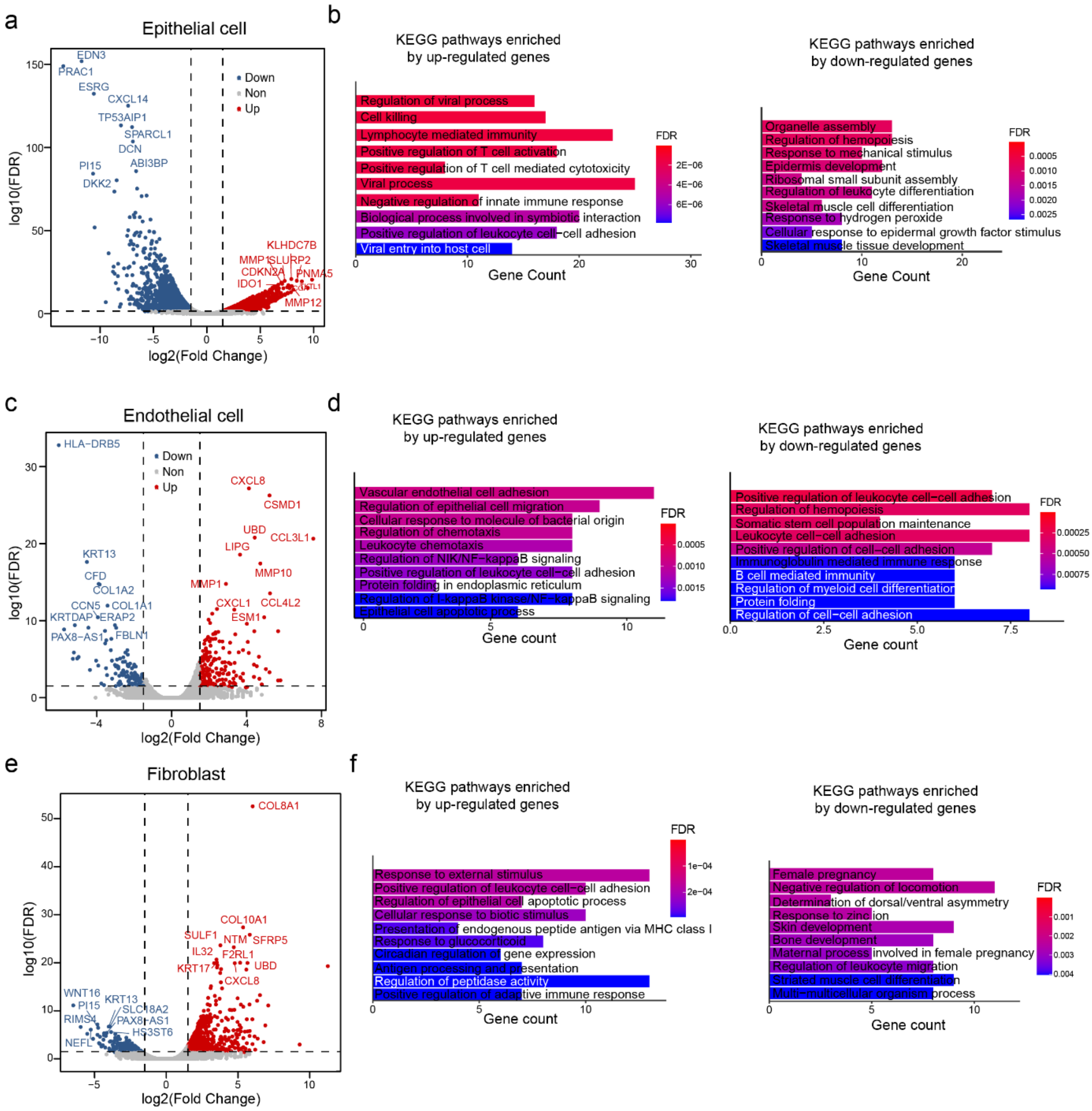

Supplementary Fig. 2 Differentially expressed genes and enriched KEGG pathways of non-immune cells between tumor and NAT samples, including epithelial cells and endothelial cells.

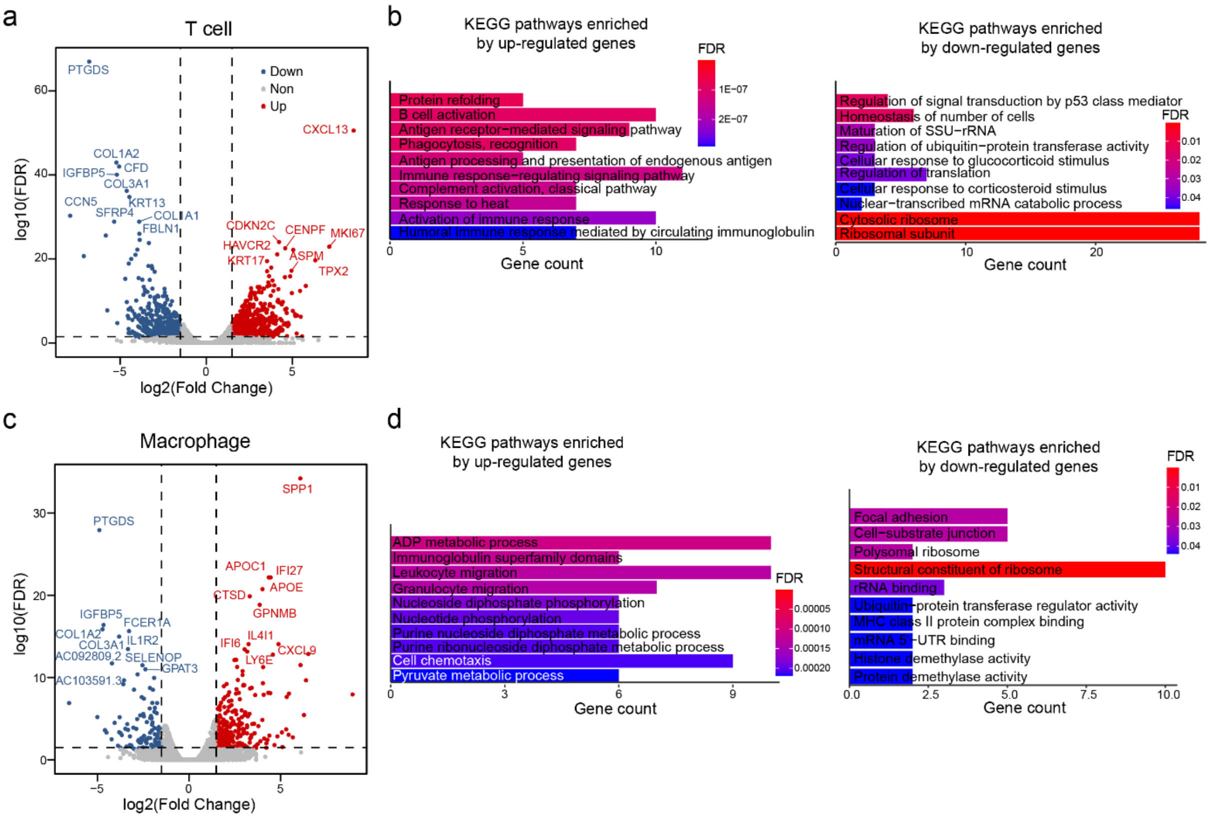

Supplementary Fig. 3 Differentially expressed genes and enriched KEGG pathways of immune cells between tumor and NAT samples, including T cells and macrophage.

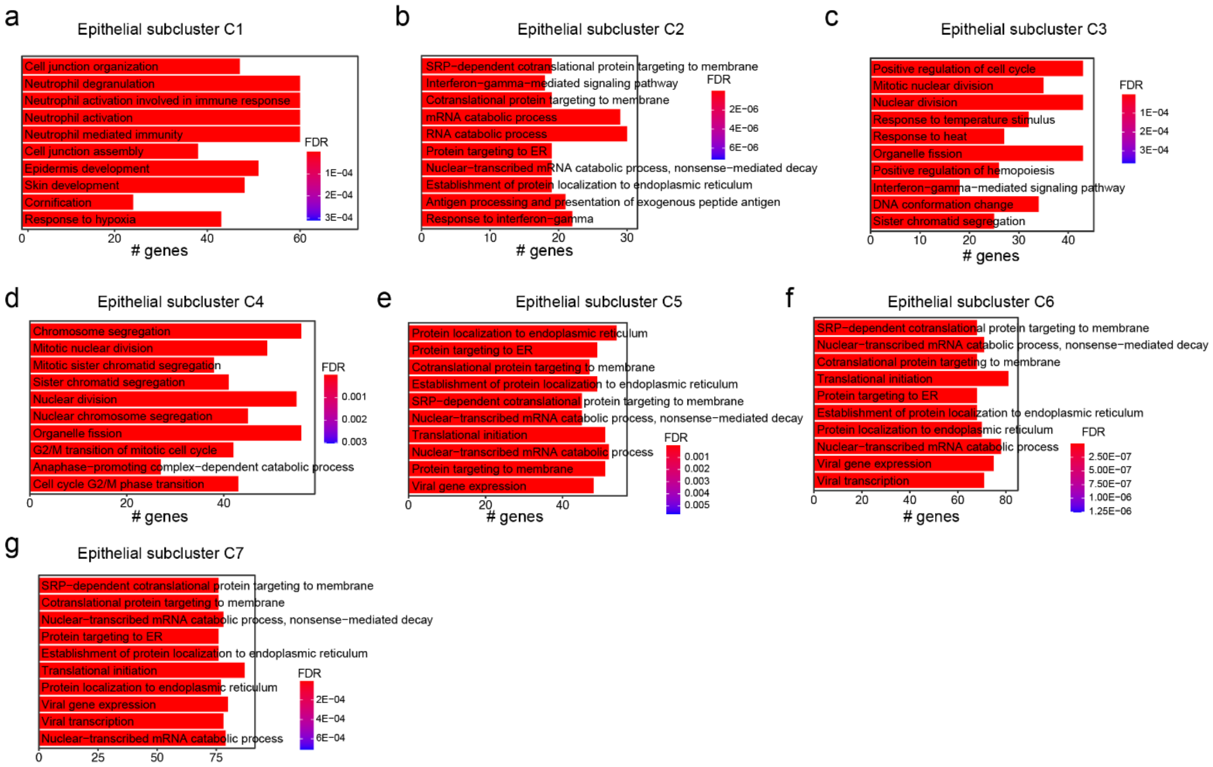

Supplementary Fig. 4 Enriched GO biological processes by marker genes in epithelial subclusters.

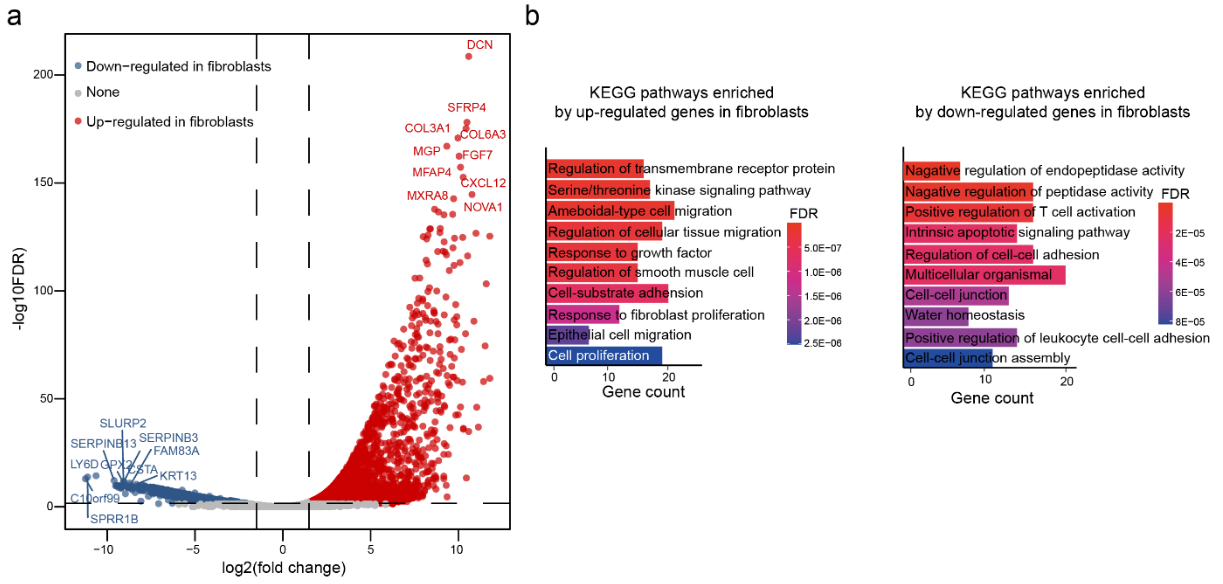

Supplementary Fig. 5 Comparison between tumor cells and tumor-derived fibroblasts. a Volcano plot showing the differentially expressed genes between tumor cells and tumor-derived fibroblasts. **b** KEGG pathways enriched by up-regulated and down-regulated genes in tumor-derived fibroblasts.

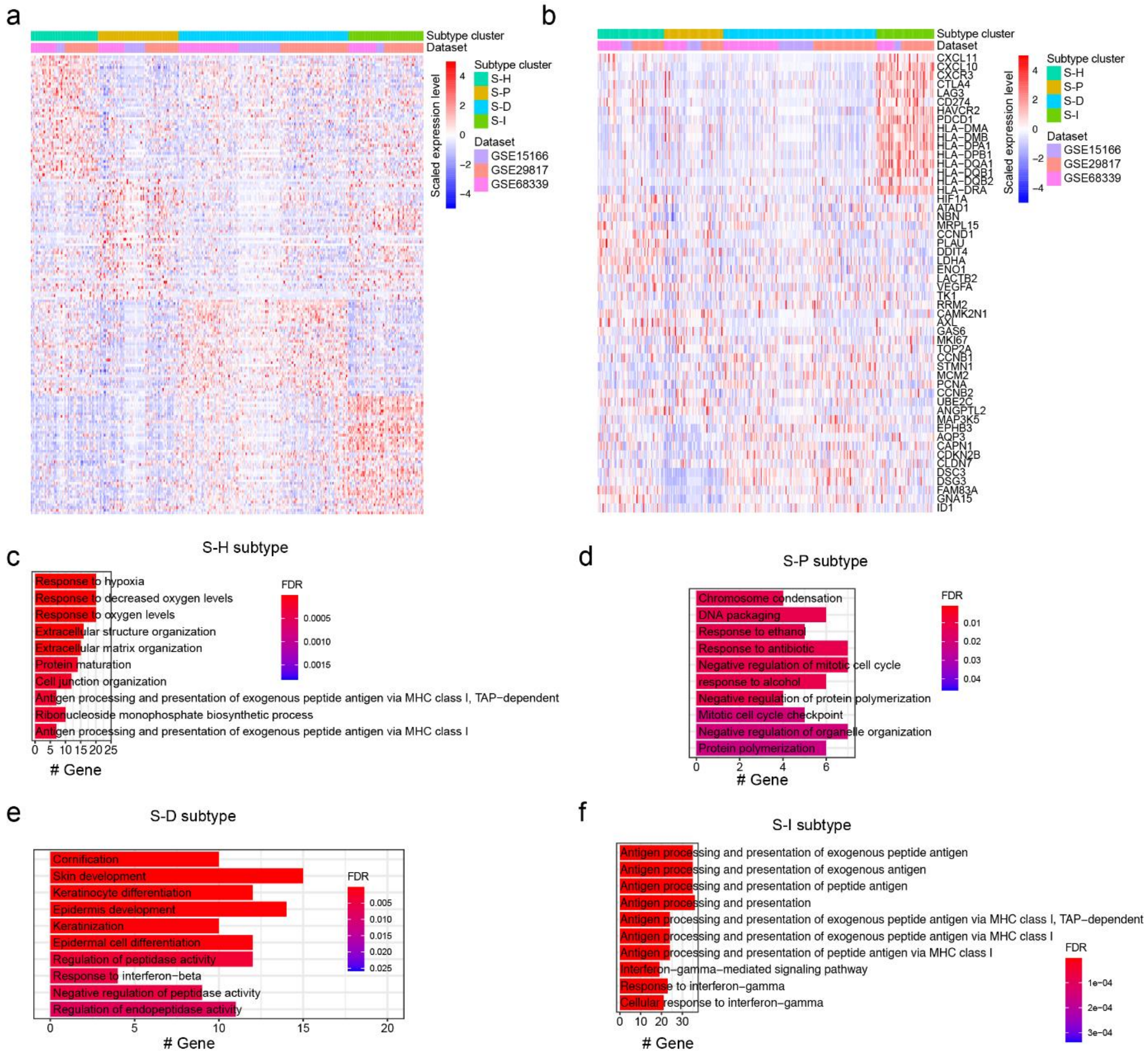

Supplementary Fig. 6 The subtype classification in GEO datasets. a Heatmap shows the clustering of CC samples from three GEO datasets. **b** Heatmap shows the expression patterns of signature genes in different subtypes. The enriched biological processes by DEGs in each subtype in GEO datasets (**c**-**f**).

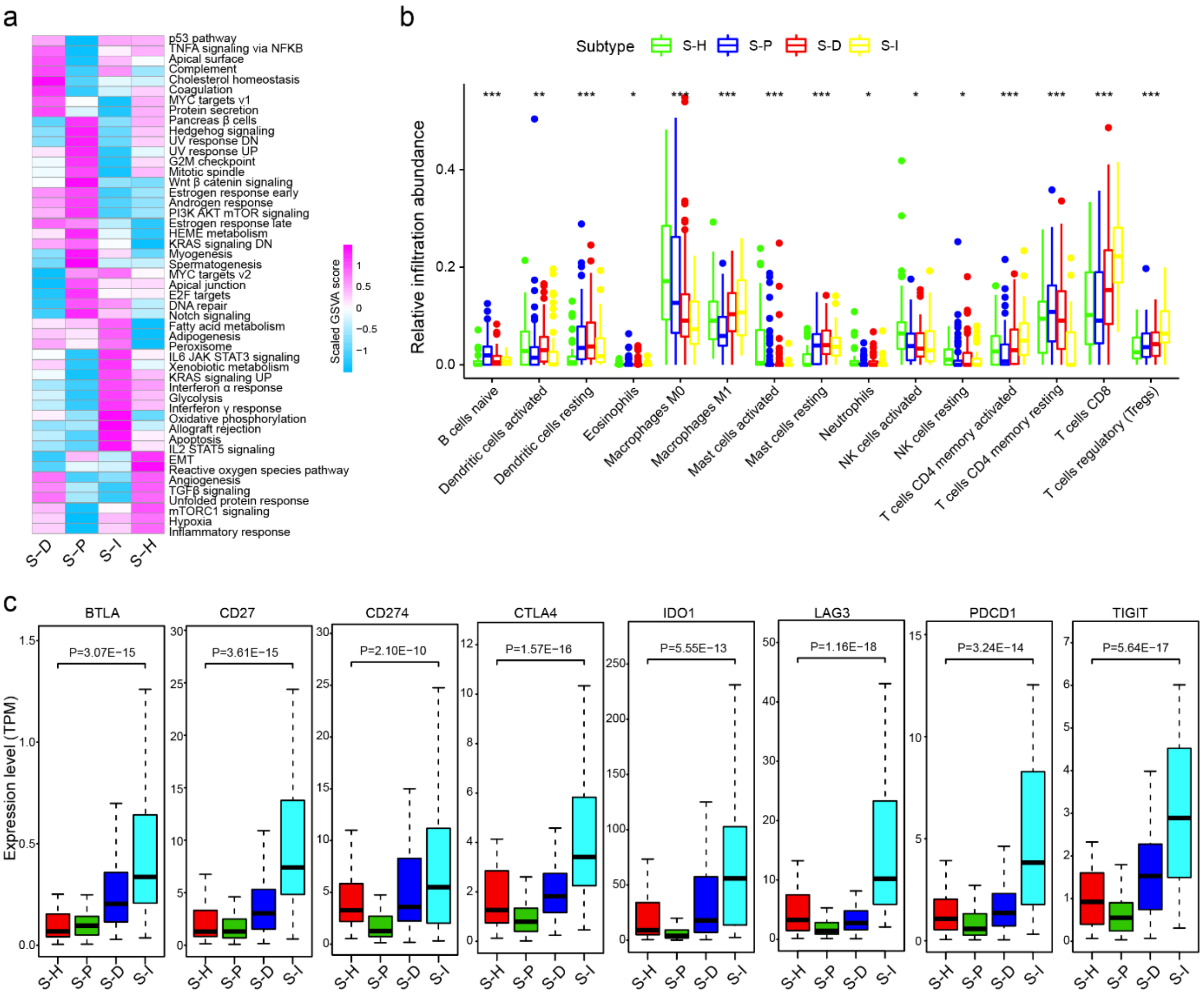

Supplementary Fig. 7 Differences among the four CC subtypes. a The relative scores of 50 hallmarks among the four CC subtypes. **b** Comparison of relative abundance of immune cell infiltration between different CC subtypes. **c** Comparison of expression levels of immune checkpoint genes between different CC subtypes. $^{*}P < 0.05$, $^{**}P < 0.01$, $^{***}P < 0.001$.

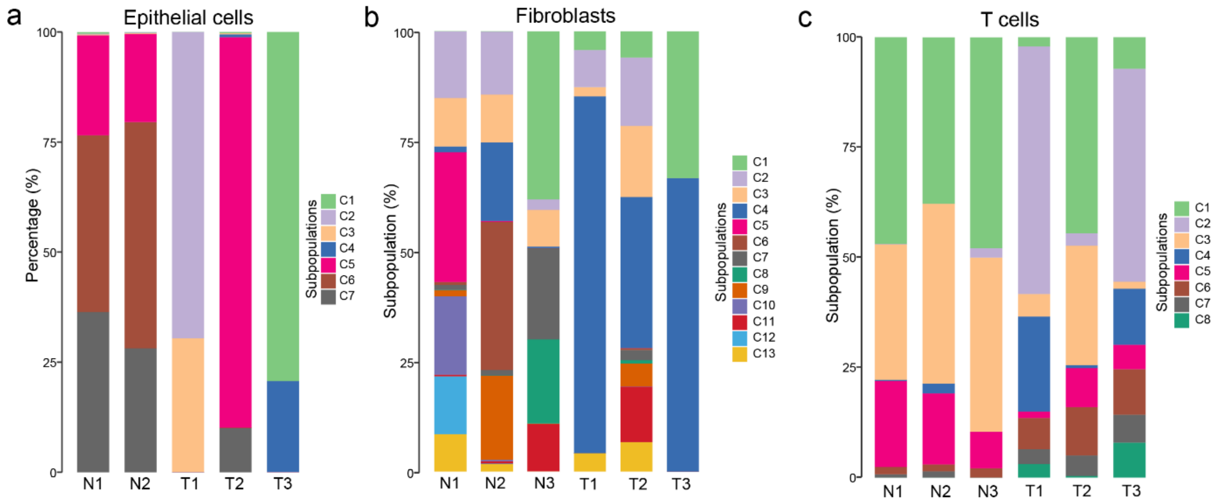

Supplementary Fig. 8 The percentages of subpopulations of epithelial cells (a), fibroblasts (b), and T cells (c) in CC cancer and paired NAT samples.

Supplementary methods

Gene set variation analysis of hallmarks

The gene sets of 50 hallmarks were retrieved from the Molecular Signature Database (MSigDB)[1]. The Gene Set Variation Analysis (GSVA)[2] algorithm was then employed to evaluate the variation of hallmark gene sets in each sample. In each sample, the expression matrix of genes in each hallmark gene set was subjected to an unsupervised calculation to infer the hallmark scores of each sample.

Abundance estimation of infiltrating immune cell

The CIBERSORT[3] method was applied to the gene expression matrix to estimate relative abundance of different immune cell types in each sample. Pre-defined gene signature of each immune cell type was utilized to deconvolve the gene expression in each sample. In particular, the LM22 immune cell signature was used, which was demonstrated to differentially express in certain cell types compared to the other cells. The LM22 signature contains gene signatures of 22 different immune cell types.

Supplementary references

1. Liberzon, A. *et al.* The Molecular Signatures Database Hallmark Gene Set Collection. *Cell Syst.* **1**, 417–425 (2015).
2. Hänzelmann, S., Castelo, R. & Guinney, J. GSVA: Gene set variation analysis for microarray and RNA-Seq data. *BMC Bioinformatics* **14**, 7 (2013).
3. Newman, A. M. *et al.* Robust enumeration of cell subsets from tissue expression profiles. *Nat. Methods* **12**, 453–457 (2015).
