# Supplementary material for: Single-cell transcriptomics reveals cellular heterogeneity and molecular stratification of cervical cancer
Source: Commun Biol. 2022 Nov 10;5:1208. doi: 10.1038/s42003-022-04142-w (PMC9649750; doi:10.1038/s42003-022-04142-w)
Supplement: Supplementary file 2 — Description of Additional Supplementary Files [file 42003_2022_4142_MOESM2_ESM.pdf]

Description of Additional Supplementary Files

File name: Supplementary Data 1

Description: Overall CAN levels in each epithelial cell. Related to Fig. 2b.

File name: Supplementary Data 2

Description: Gene expression differences between tumor and NAT fibroblast. Related to Fig. 3d.

File name: Supplementary Data 3

Description: Gene expression differences between iCAF and myCAF cells. Related to Fig. 3h.

File name: Supplementary Data 4

Description: Gene expression differences across CD8+ T cell subclusters. Related to Fig. 4c.
