# Supplementary material for: Single-cell transcriptomics reveals cellular heterogeneity and molecular stratification of cervical cancer
Source: Commun Biol. 2022 Nov 10;5:1208. doi: 10.1038/s42003-022-04142-w (PMC9649750; doi:10.1038/s42003-022-04142-w)
Supplement: Supplementary file 7 — Reporting Summary [file 42003_2022_4142_MOESM7_ESM.pdf]

nature portfolio

Corresponding author(s): Shengli Li

Last updated by author(s): Oct 5, 2022

Reporting Summary

Nature Portfolio wishes to improve the reproducibility of the work that we publish. This form provides structure for consistency and transparency in reporting. For further information on Nature Portfolio policies, see our Editorial Policies and the Editorial Policy Checklist.

Statistics

For all statistical analyses, confirm that the following items are present in the figure legend, table legend, main text, or Methods section.

n/a	Confirmed	
☐	☒	The exact sample size (*n*) for each experimental group/condition, given as a discrete number and unit of measurement
☐	☒	A statement on whether measurements were taken from distinct samples or whether the same sample was measured repeatedly
☐	☒	The statistical test(s) used AND whether they are one- or two-sided<br>*Only common tests should be described solely by name; describe more complex techniques in the Methods section.*
☐	☒	A description of all covariates tested
☐	☒	A description of any assumptions or corrections, such as tests of normality and adjustment for multiple comparisons
☐	☒	A full description of the statistical parameters including central tendency (e.g. means) or other basic estimates (e.g. regression coefficient) AND variation (e.g. standard deviation) or associated estimates of uncertainty (e.g. confidence intervals)
☐	☒	For null hypothesis testing, the test statistic (e.g. *F*, *t*, *r*) with confidence intervals, effect sizes, degrees of freedom and *P* value noted<br>*Give P values as exact values whenever suitable.*
☒	☐	For Bayesian analysis, information on the choice of priors and Markov chain Monte Carlo settings
☒	☐	For hierarchical and complex designs, identification of the appropriate level for tests and full reporting of outcomes
☐	☒	Estimates of effect sizes (e.g. Cohen's *d*, Pearson's *r*), indicating how they were calculated

Our web collection on statistics for biologists contains articles on many of the points above.

Software and code

Policy information about availability of computer code

Data collection	No software was used for data collection.
Data analysis	Cell Ranger (version 3.0.1), Seurat R package (version 4.0), Scrublet software (version 0.2.2), SingleR R package (version 1.4.1), inferCNV software (https://github.com/broadinstitute/infercnv), Monocle2 R package (version 2.8.0), clusterProfiler R package (version 4.1), pySCENIC (version 0.10.2)

For manuscripts utilizing custom algorithms or software that are central to the research but not yet described in published literature, software must be made available to editors and reviewers. We strongly encourage code deposition in a community repository (e.g. GitHub). See the Nature Portfolio guidelines for submitting code & software for further information.

Data

Policy information about availability of data

All manuscripts must include a data availability statement. This statement should provide the following information, where applicable:

- Accession codes, unique identifiers, or web links for publicly available datasets
- A description of any restrictions on data availability
- For clinical datasets or third party data, please ensure that the statement adheres to our policy

Single-cell RNA sequencing gene expression data generated in this study has been deposited in the ArrayExpress database with accession of E-MTAB-11948 (https://

www.ebi.ac.uk/biostudies/arrayexpress/studies/E-MTAB-11948). Any other data are available from the corresponding author on reasonable request. Software and resources used for analysis and plotting are described in each method section.

Human research participants

Policy information about studies involving human research participants and Sex and Gender in Research.

Reporting on sex and gender	Only human biospecimens were used in this study. Cervical cancer and non-cancer samples were derived from female patients. All informed contents were acquired from patients
Population characteristics	Not relevant in our study.
Recruitment	Not relevant in our study.
Ethics oversight	The ethics committee of the Obstetrics and Gynecology Hospital of Fudan University

Note that full information on the approval of the study protocol must also be provided in the manuscript.

Field-specific reporting

Please select the one below that is the best fit for your research. If you are not sure, read the appropriate sections before making your selection.

☒ Life sciences ☐ Behavioural & social sciences ☐ Ecological, evolutionary & environmental sciences

For a reference copy of the document with all sections, see nature.com/documents/nr-reporting-summary-flat.pdf

Life sciences study design

All studies must disclose on these points even when the disclosure is negative.

Sample size	Six samples, including three cervical cancer and three paired non-cancer cervical samples, were used to perform single-cell RNA sequencing. In the deconvolution analysis of bulk RNA-seq data, 253 samples from the TCGA CESC cohort and 340 samples from the GEO database (GSE15166, GSE29617, and GSE68335) were used.
Data exclusions	No data exclusion.
Replication	All data analyses are reproducible.
Randomization	Randomization is not relevant to our study.
Blinding	Blinding is not relevant to our study.

Reporting for specific materials, systems and methods

We require information from authors about some types of materials, experimental systems and methods used in many studies. Here, indicate whether each material, system or method listed is relevant to your study. If you are not sure if a list item applies to your research, read the appropriate section before selecting a response.

Materials & experimental systems

n/a	Involved in the study	
☒	☐	Antibodies
☒	☐	Eukaryotic cell lines
☒	☐	Palaeontology and archaeology
☒	☐	Animals and other organisms
☒	☐	Clinical data
☒	☐	Dual use research of concern

Methods

n/a	Involved in the study	
☒	☐	ChIP-seq
☒	☐	Flow cytometry
☒	☐	MRI-based neuroimaging
